# Unraveling IFN-I response dynamics and TNF crosstalk in the pathophysiology of systemic lupus erythematosus

**DOI:** 10.3389/fimmu.2024.1322814

**Published:** 2024-03-26

**Authors:** Laura C. Van Eyndhoven, Eleni Chouri, Catarina I. Matos, Aridaman Pandit, Timothy R. D. J. Radstake, Jasper C. A. Broen, Abhyudai Singh, Jurjen Tel

**Affiliations:** ^1^ Laboratory of Immunoengineering, Department of Biomedical Engineering, Eindhoven University of Technology, Eindhoven, Netherlands; ^2^ Institute for Complex Molecular Systems (ICMS), Eindhoven University of Technology, Eindhoven, Netherlands; ^3^ Center for Translational Immunology, Department of Immunology, University Medical Center Utrecht, Utrecht University, Utrecht, Netherlands; ^4^ Department of Rheumatology and Clinical Immunology, University Medical Center Utrecht, Utrecht University, Utrecht, Netherlands; ^5^ Regional Rheumatology Center, Máxima Medical Center, Eindhoven and Veldhoven, Eindhoven, Netherlands; ^6^ Department of Electrical and Computer Engineering, University of Delaware, Newark, DE, United States

**Keywords:** type I interferon, plasmacytoid dendritic cells, systemic lupus erythematosus, heterogeneity, droplet-based microfluidics, ODE modeling, single-cell analysis

## Abstract

**Introduction:**

The innate immune system serves the crucial first line of defense against a wide variety of potential threats, during which the production of pro-inflammatory cytokines IFN-I and TNFα are key. This astonishing power to fight invaders, however, comes at the cost of risking IFN-I-related pathologies, such as observed during autoimmune diseases, during which IFN-I and TNFα response dynamics are dysregulated. Therefore, these response dynamics must be tightly regulated, and precisely matched with the potential threat. This regulation is currently far from understood.

**Methods:**

Using droplet-based microfluidics and ODE modeling, we studied the fundamentals of single-cell decision-making upon TLR signaling in human primary immune cells (n = 23). Next, using biologicals used for treating autoimmune diseases [i.e., anti-TNFα, and JAK inhibitors], we unraveled the crosstalk between IFN-I and TNFα signaling dynamics. Finally, we studied primary immune cells isolated from SLE patients (n = 8) to provide insights into SLE pathophysiology.

**Results:**

single-cell IFN-I and TNFα response dynamics display remarkable differences, yet both being highly heterogeneous. Blocking TNFα signaling increases the percentage of IFN-I-producing cells, while blocking IFN-I signaling decreases the percentage of TNFα-producing cells. Single-cell decision-making in SLE patients is dysregulated, pointing towards a dysregulated crosstalk between IFN-I and TNFα response dynamics.

**Discussion:**

We provide a solid droplet-based microfluidic platform to study inherent immune secretory behaviors, substantiated by ODE modeling, which can challenge the conceptualization within and between different immune signaling systems. These insights will build towards an improved fundamental understanding on single-cell decision-making in health and disease.

## 1 Introduction

The innate immune system serves the crucial first line of defense against a wide variety of potential threats. Accordingly, the production of pro-inflammatory cytokines upon toll-like receptor (TLR) signaling, and other pathogen recognition receptors, requires a finetuned balance between rapid yet robust immune activation, while preventing chronic and out-of-control inflammation (1–5). In an attempt to capture immune secretory behaviors in rather simplified models, population-level studies have suggested highly constrained models, where target gene responses are subjected to tight epigenetic and transcriptional regulation (6–8). In contrast, at the single-cell level, TLR effector responses exhibit high variability characterized by all-or-nothing cellular decision-making (9–15). This heterogeneity is thought to reflect complex transcriptional regulation, characterized by dynamic transcription factor signaling (16, 17) as well as diverse genomic architecture (18) and immune quorum sensing/licensing (19–21).

Over the past decades, the optimization and utilization of single-cell approaches allowed to further unravel the fundamentals of cellular decision-making during immune responses (22). Besides, single-cell RNA sequencing (scRNA-seq), single-molecule RNA fluorescence *in situ* hybridization (smRNA-FISH), single-cell quantitative PCR, and numerous microfluidic approaches have revealed their revolutionary power to assess both cellular phenotypes as well as the functional, in this case secretory behaviors of single cells (11, 23–25). In particular, droplet-based microfluidics allowed for the activation of single cells, which provides advantages over single-cell analysis of bulk-activated cells by revealing cell-intrinsic behaviors independent from the paracrine and juxtacrine signaling (26). This method allowed for the characterization of three distinct cell fates driving type I IFN (IFN-I) response dynamics (reviewed in (23). Upon homogeneous activation with synthetic viral nucleotides only a fraction of 1-3% of the total population will initiate IFN-I production, referred to as the first responders. Via paracrine signaling, these first responders initiate additional IFN-I production in a second, much larger fraction of the total population (10-40%), which are referred to as the second responders. This leaves the majority (60-90% of the total population) of seemingly identical cells left unresponsive, meaning this fraction will not produce IFN-Is despite being infected or activated via paracrine signaling. Altogether, the rise of single-cell technologies enabled numerous breakthroughs related to the fundamentals of cellular decision-making in various immune signaling systems. Examples include the cellular decision-making during T helper differentiation upon varying IFNγ and interleukin 4 (IL-4) inputs (27), all-or-nothing decision making during nuclear factor kB (NF-kB) signaling controlled by epigenetic licensing (28), among hundreds of key immune genes that are bimodally expressed across cells (29).

While most, if not all, immune signaling systems are inherently complex by nature, most studies have focused on individual signaling systems and components (e.g., IFNγ, IL-4, TNFα, NF-kB, IFN-I). *In vivo*, however, these systems are intertwined into complex interplays. Therefore, studying both IFN-I and TNFα secretory behaviors can reveal insights into the interplay between the two cytokines, better reflecting the *in vivo* situation, thereby allowing for better translatability of results. In the context of autoimmune diseases, such as systemic lupus erythematosus (SLE) and rheumatoid arthritis (RA), the crosstalk between IFN-I and TNFα is hypothesized to be of crucial importance, as literature suggests an inhibitory effect of TNFα on IFN-Is, and vice versa (30, 31). Multiple drugs for treating auto-immune disease are focused on primarily blocking IFN-I or TNFα (e.g., with Janus kinase inhibitors (JAKi) and anti-TNFα antibodies), which might lead to undesired up-/dysregulation of the untargeted one (30). In contrast to the hypothesis on their inhibitory effects, other studies highlighted a cooperative role for IFN-I and TNFα, while IFN-Is potentiate the inflammatory function of TNFα by priming chromatin to prevent the silencing of target genes that encode inflammatory molecules (32). In short, the crosstalk between IFN-I and TNFα remains far from understood.

In this study, we characterized single-cell decision-making in human primary immune cells upon TLR stimulation using droplet-based microfluidics to unravel the single-cell IFN-I and TNFα secretory behaviors, to provide insights into their regulation and crosstalk. We compared cellular decision-making in peripheral blood mononuclear cells (PBMCs) isolated from 23 healthy controls with PBMCs isolated from 8 SLE patients, which showed remarkable differences. This allowed us to uncover the intrinsic behaviors of individual cells, which appears of crucial importance to understand the fundamentals of immune signaling systems, both in health and disease (23). In the context of autoimmune diseases, we demonstrated that blocking TNFα signaling using anti-TNFα antibodies leads to upregulated IFN-I signaling dynamics, while blocking IFN-I-mediated paracrine signaling using JAKi inhibits TNFα production. Together, these novel insights pave the way to a better fundamental understanding of single-cell decision-making and the interplay between immune signaling systems, which could potentially get translated into improved immune treatment strategies targeting pro-inflammatory cytokine signaling.

## 2 Materials and methods

### 2.1 SLE patients

An identification cohort consisting of patients with SLE (n = 8) as well as healthy controls (HCs; n = 23) were studied. All patients provided informed written consent approved by the local institutional medical ethics review boards prior to inclusion in this study (NL47151.041.13). Samples of patients and HCs were obtained in the clinics of the University Medical Center Utrecht. Patients with SLE (n = 8) fulfilled their classification criteria (33). PBMCs were isolated from whole blood using density gradient centrifugation and frozen. Prior to the use of PBMCs in experiments, cells were thawed and rested for 2 hours in RPMI supplemented with 2% human serum (pooled; Sanquin) and 1% antibiotics (penicillin-streptomycin) at 37 degrees Celsius. HCs and SLE patients were not age and sex matched.

### 2.2 Cell isolation and culture

For patient-independent experiments, human primary immune cells were isolated from buffy coats of healthy donors (Sanquin), according to institutional guidelines and after informed consent per the Declaration of Helsinki, via Lymphoprep (Stemcell Technologies, 07861) density gradient centrifugation. Peripheral blood mononuclear cells (PBMCs) were washed thrice with phosphate-buffered saline (PBS, Thermo Fisher Scientific, 20012027) supplemented with 0.6 w/v% sodium citrate dehydrate tri-basic and 0.01 w/v% bovine serum albumin (Sigma Aldrich, C8532; A9418). Plasmacytoid dendritic cells (pDCs) were isolated using magnet-activated cell sorting (MACS) by positive selection using the CD304 Microbeat Kit (Miltenyi Biotec, 130-090-532), according to manufacturer’s instructions. Purity was assessed by 20 minutes staining at 4 degrees Celsius in PBS supplemented with 0.5% bovine serum albumin (later referred to as PBA) using FITC-labeled anti-CD123 and APC-labeled anti-CD303. When indicated, pDCs were rested overnight in U-bottom well plates in 100 uL X-Vivo 15 cell culture medium (Lonza), supplemented with 2% human serum (pooled; Sanquin), 1% antibiotics (penicillin-streptomycin), and 20 ng/mL interleukin 3 (IL-3), at a density of 25.000-100.000 cells per well.

### 2.3 Soft lithography and microfluidic setup

Microfluidic devices were fabricated with polydimethylsiloxane (PDMS) base and curing agent at a ratio of 10:1 (Sylgard 184; Sigma-Aldrich, 101697). The PDMS mix was poured onto a master silicon wafer and cured at 65 degrees Celsius for 3 hours. Both the surface of the devices and the glass slides were OH-terminated by exposure to plasma (Emitech K1050X) and bonded to yield closed microchannels. Finally, channels were treated with 2% silane in fluorinated HFE-7500 3M Novec (Fluorochem, 051243). Liquids were dispensed from syringes driven by computer-controlled pumps (Nemesys, Cetoni GmbH). 2.5 v/v% Pico-Surf surfactant (Sphere Fluidics, C024) in fluorinated HFE-7500 3M Novec was used for the oil inlet, whereas mineral oil was used for the two aqueous phases. The cell suspension and stimulus suspension were loaded onto the microfluidic device by using the Tip-Loading method, as described elsewhere (34).

### 2.4 Bulk activation assay in microwells

Freshly isolated PBMCs or pDCs were incubated in 100 μL per 10^6^ cells PBA containing the TNFα and IFNα Cytokine Catch Reagent (Miltenyi Biotec, 130-092-605) at 4 degrees Celsius for 20 minutes. Next, cells were washed and resuspended X-Vivo 15 cell culture medium (Lonza), supplemented with 2% human serum (pooled; Sanquin), 1% antibiotics (penicillin-streptomycin), at 25.000 cells per 100 uL in U-bottom microwell plates. Regarding all experiments in which cytokine production was assessed by intracellular cytokine stainings, cells were not pre-incubated with Cytokine Catch Reagent, but directly transferred to the microwells upon isolation.

### 2.5 Single-cell activation assay in picoliter droplets

Single-cell encapsulation was achieved at a cell concentration of 2.6×10^6^ cells/mL in 92 pL droplets on average, as described elsewhere (14, 26). Droplets were produced at flow rates of 900 μL/h for the oil phase and 300 μL/h for the aqueous phases. Single-cell encapsulation and droplet production were carefully monitored using a microscope (Nikon) at 10x magnification and a high-speed camera. The droplet emulsion was collected in Eppendorf tubes with punched holes to allow gas exchange, covered with culture medium to protect the emulsion from evaporation, and incubated at 37 degrees Celsius and 5% CO2. After 18 hours of incubation, the droplets were de-emulsified by adding 100 uL 20 v/v% 1H,1H,2H,2H-Perfluoro-1-octanol (Sigma Aldrich, 370533) in HFE-7500.

### 2.6 Antibody staining

Cells were washed with PBS and dead cells were stained with Zombie Green fixable viability dye (BioLegend, 423111), 1:10.000 in PBS, 100 uL) at 4 degrees Celsius for 20 minutes. Next, cells were washed and incubated with antibodies against surface proteins in 50 μL PBA at 4 degrees Celsius for 20 minutes. Regarding the time-course experiments, after each timepoint, cells were fixed with Cytofix/Cytoperm solution (BD Biosciences, 554714) at 4 degrees Celsius for 20 minutes and kept at 4 degrees Celsius upon measuring.

### 2.7 Flow cytometry

Acquisition was performed in PBA on FACS Aria (BD Biosciences). Flow cytometry data were analyzed using FlowJo X (Tree Star). FMO stainings served as controls for gating strategy. For the gating strategy, the readers are referred to **Supplementary Figure 1**.

### 2.8 ELISA analysis

Samples were collected at various time points to quantify TNFα and IFNα production by ELISA (BioLegend 430204, 446404) according to the manufacturer’s instructions, at limit of quantification (LOQ) of 7.8 pg/mL and 12.5 pg/mL for TNFα and IFNα, respectively.

### 2.9 ODE modeling

To model the IFN-I system dynamics, we consider a small fraction of first responders that are activated in response to the stimulus. IFN-I secreted from these first responders activates a larger fraction of second responders via paracrine signaling. We assume across stimuli and dosages that a fixed fraction of 1% of cells are first responders, whereas the second responder fraction can vary, however, originating from a fixed pool of maximum 50% of potential second responders that become activated upon reaching an activation threshold. The remaining cells are nonresponders.

The model is described by the following differential equations:


$$\frac{df_{1}}{dt}=k_{on}\left(fr-f_{1}\right)-k_{off}f_{1}$$

$$\frac{df_{2}}{dt}=k_{on}\frac{{\left[IFN\right]}^{H}}{T^{H}+{\left[IFN\right]}^{H}}(sr-f_{2})-k_{off}f_{2}$$

$$\frac{d[IFN]}{dt}=k_{f}(f_{1}+f_{2})-d_{f}[IFN]$$

where we set the fraction of first responders and the second responder pool as fr=0.01 and sr=0.5 , respectively. The first two equations describe the activated first-responder $f_{1}$ , and actual second responders $f_{2}\;$ over time with $f_{1}+f_{2}$ being the total fraction of IFN α -positive cells. The third equation captures the build of IFN α concentration [IFN] with each activated cell secreting it at a rate $k_{f}$ , and $d_{f}\;$ , referring to the IFN α consumption and degradation rate, respectively. First responders activate with rate $k_{on}$ and tun off with rate $k_{off}$ , while second responders are activated at an interferon-dependent rate $k_{on}\frac{{\left[IFN\right]}^{H}}{T^{H}+{\left[IFN\right]}^{H}}\;$ and turn off with the same rate as first-responders. Here the positive constant T can be interpreted as the interferon threshold for activation of second responders and H is the Hill coefficient, capturing the quantification of the degree of interaction between ligand [i.e., IFN-I] and binding sites [i.e., IFNAR]. All model fits were done by performing least square fitting with Microsoft Excel Solver Toolbox using the Generalized Reduced Gradient (GRC) nonlinear method.

### 2.10 Data analysis and statistics

Analysis and data visualization was performed using PRISM for windows version 9 (GraphPad). For statistical analysis, Student’s t-test, and Mixed-effects analysis followed by a Bonferroni’s multiple comparisons test were performed.

## 3 Results

### 3.1 TLR-induced cellular decision-making in human primary immune cells

**Figure 1:**
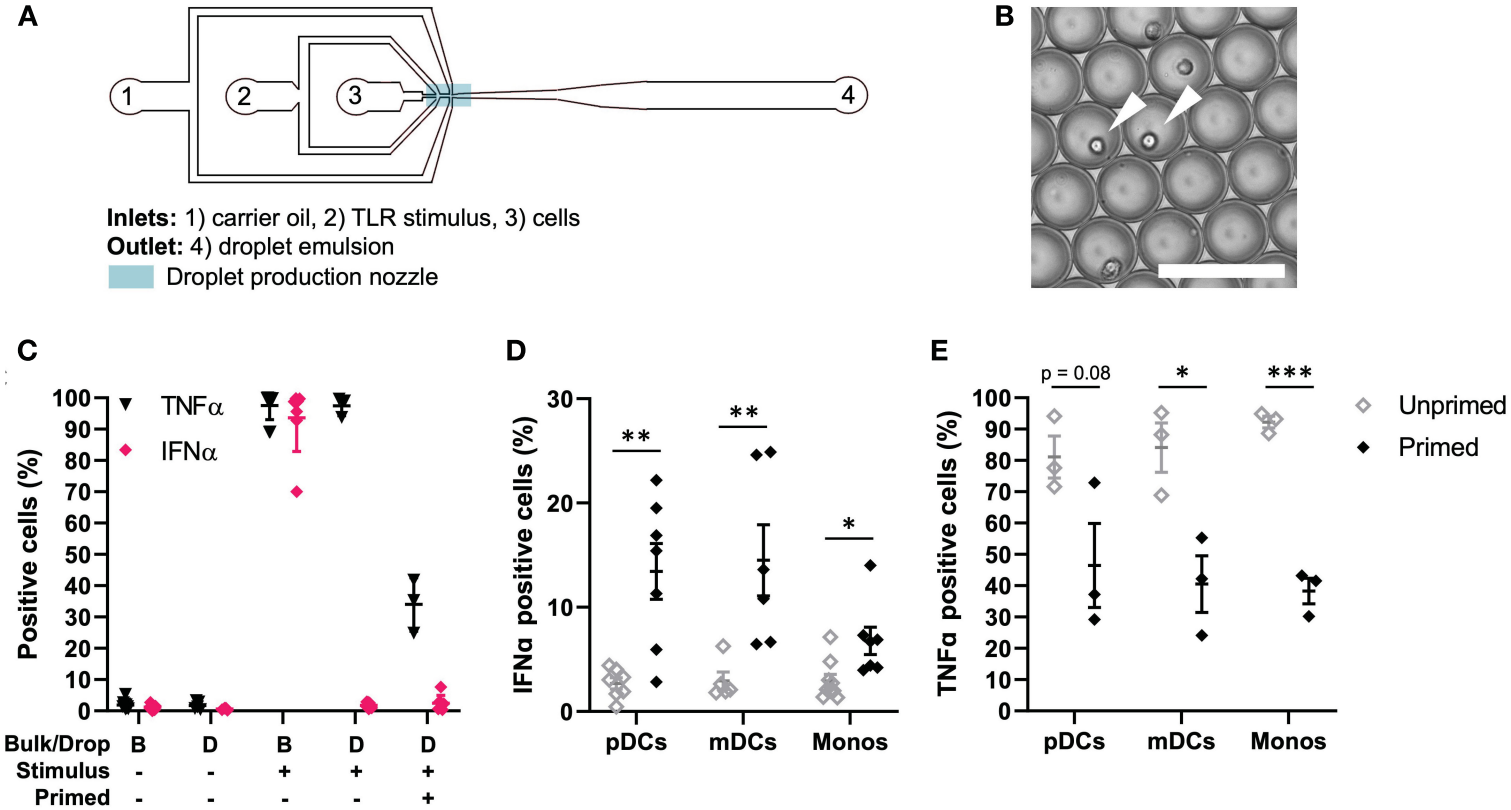
TLR-induced cellular decision-making in human primary immune cells. **(A)** Schematic overview of microfluidic chip for single-cell encapsulation in water-in-oil droplets. **(B)** Microscopy image of droplet emulsion with single-encapsulated cells, indicated with white arrows. Scale bar equals 100 μm. **(C)** Human PBMCs were isolated from buffy coats, primed with 500 U/mL IFNβ or left untreated. Next, cells were coated with Cytokine Catch Reagents for IFNα and TNFα, followed by TLR activation with 5 or 50 μg/mL R848 for bulk and droplets conditions, respectively. After incubation, cells were retrieved from droplets by de-emulsification and analyzed by flow cytometry. Depicted are the percentages of positive cells, including mean ± SEM; n = 3. **(D)** IFNα positive cells upon single-cell activation in droplets. Depicted are the percentages of different types of immune cells: plasmacytoid dendritic cells (pDCs), myeloid dendritic cells (mDCs), and monocytes (monos), including mean ± SEM. n = 9; Student’s t-test *p< 0.05, **p< 0.01. **(E)** TNFα positive cells upon single-cell activation in droplets. Depicted are the percentages of different types of immune cells, as in **(D)**; mean ± SEM. n = 3; Student’s t-test *p< 0.05, ***p< 0.001.

To obtain insights into the single-cell decision-making in human primary immune cells, we utilized our droplet-based microfluidic platform for single-cell activation (26, 34). Upon single-cell encapsulation in droplets, the effects of paracrine signaling can be eliminated, while secreted molecules cannot diffuse from droplet to droplet. Therefore, this technique allows for an elegant way to study cell intrinsic capabilities to respond to a stimulus. Accordingly, we and others found that upon single-cell activation of bone-marrow-derived mouse dendritic cells and in human primary pDCs, only very small fractions of cells can produce IFN-Is (1-3% of the total population), which have been referred to as precocious cells and first responders (11, 14, 26). In turn, paracrine signaling induces IFN-I production in much larger fractions of cells (10-40%), which we like to refer to as second responders, given that they need a secondary input (paracrine signaling) over the first, primary input [pathogenic ligand; reviewed in (23)]. Previously, we showed that priming with conditioned media obtained from activated pDCs was able to induce second responders upon single-cell activation in droplets, after which we identified IFNβ as being the most potent priming cytokine (26). For this study, we first aimed to explore whether we could observe the phenomenon of so-called first, second and non-responders in other IFN-I producing human primary immune cells. Human primary peripheral blood mononuclear cells (PBMCs) were isolated from healthy donors. Upon isolation, cells were either primed with 500 U/mL IFNβ (mimicking paracrine signaling) or left unprimed, followed by encapsulation in water-in-oil droplets using a microfluidic chip (**Figure 1A**). The priming concentration was previously optimized (26). Monodispersity and single-cell encapsulation was carefully monitored during droplet generation (**Figure 1B**). Prior to encapsulation, cells were coated with cytokine catch reagents, for both TNFα and IFNα, to monitor single-cell cytokine production. In droplet conditions, this approach allows for the monitoring of single-cell cytokine production as the produced cytokines are only able to bind to cells that have actually produced it (26). In bulk conditions, this approach can be considered as an important internal control to assess proper activation of PBMCs, resulting in numbers of positive cells up to 100% as a consequence of cytokine diffusion, thereby saturating the catch reagents on surrounding cells (14).

Next, we assessed cytokine secretion upon single-cell activation in total PBMCs. As expected, both in bulk and upon single-cell encapsulation in droplets, the cells do not secrete TNFα nor IFNα when left unstimulated (**Figure 1C**). Once activated in bulk with TLR7/8 ligand R848, numbers of positive cells reach up to 100%, as described before. Interestingly, upon single-cell activation in droplets, roughly all cells can produce TNFα, whereas only very small fractions produce IFNα. Upon IFNβ priming, the number of TNFα positive cells drops to roughly 20-40%, which could be explained by the inhibitory effects of IFNβ priming on TNFα induction (30). The number of IFNα positive cells in total PBMCs only increases slightly. Therefore, we next analyzed individual immune cell subsets which are known for their ability to mass-produce IFNα upon TLR stimulation, namely pDCs (CD123^+^), myeloid dendritic cells (mDCs; CD1c^+^) and monocytes (CD14^+^) (**Supplementary Figure 1**). Interestingly, both mDCs and monocytes showed very similar results compared to pDCs. In short, upon single-cell activation in droplets, only fractions of 1-3% start producing IFNα, which are referred by literature as first responders (**Figure 1D**). In other words, these cells only need the viral input to initiate IFN-I secretion, and comprise what is referred to as the early IFN-I phase (35). Upon priming, the percentage of IFNα-producing cells increases up to 30%. This additional IFN-I production is based on receiving both the viral input and the paracrine input [i.e. IFN-I priming], which in practice can only occur after the activation of the first responders (23, 35). Finally, we analyzed TNFα production in the same immune cell subsets, again proving the inhibitory effect of IFNβ priming on TNFα production (**Figure 1E**).

Taken together, we demonstrated that the phenomenon of first, second and non-responders, related to IFN-I single-cell decision-making, is conserved across human primary immune cells. In contrast, all PBMCs have the inherent capacity to produce TNFα, which is reduced upon IFNβ priming.

### 3.2 Modeling IFN-I response dynamics upon varying TLR stimulation

**Figure 2:**
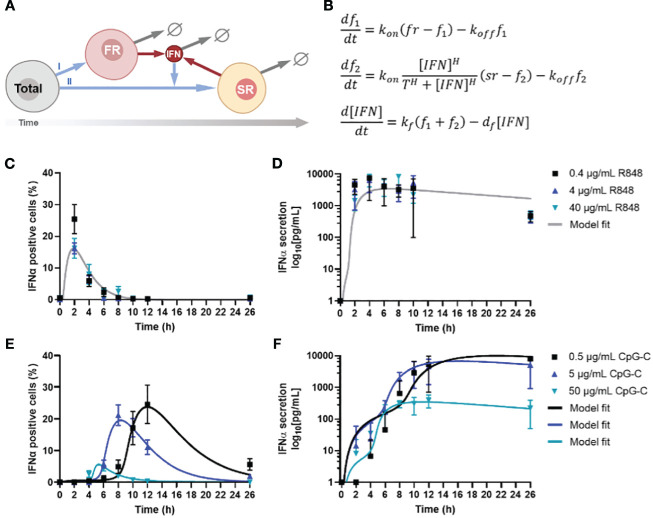
Model analysis of IFN-I response dynamics upon various TLR stimulation. **(A)** Schematic representation of IFN-I model, in which a fixed fraction of first responders (FR) originates from the total population of cells, after which they start producing IFN-Is, which induces second responders (SR) to originate from the remaining total population, leading to additional IFN-I production. The circle-backslash symbols indicate the decay of IFN-I and IFN-I-producing cells, whereas the arrows indicate the flow logic from one model component to the next. **(B)** ODE model equations. **(C)** IFNα response dynamics, represented as percentage of positive cells, upon activation with various concentrations of R848, including model fit; mean ± SEM; n = 6-9 and 5-6. **(D)** Corresponding IFNα secretion dynamics, including model fit. **(E)** IFNα response dynamics, represented as percentage of positive cells, upon activation with various concentrations of CpG-C, including model fit; mean ± SEM; n = 5-14 and 4-13. **(F)** Corresponding IFNα secretion dynamics, including model fit.

The clear distinction between first and second responders dictating IFN-I response dynamics motivated us to capture the IFN-I response dynamics in mathematical models, thereby aiming to decode the single-cell decision-making dynamics upon varying TLR stimulation. Using an ordinary differentially equation (ODE) model, we captured the 3 different cell fates [i.e., first, second, and nonresponders] and their distinct behaviors. In short, a small but fixed fraction of first responders appears from the total population upon (homogeneous) viral infection, which is independent from stimulus type, and starts to produce IFN-Is (26) (**Figure 2A**). In a paracrine fashion, this first wave of IFN-Is starts to induce additional IFN-I production in a much larger fraction of responders, referred to as second responders, which appears from the total population (minus first responders) upon viral infection and IFN-I signaling via the IFN-I receptor (IFNAR). Both fractions produce IFN-Is for only a limited amount of time, defined by a decay rate. The ODE model was defined by three equations (**Figure 2B**).

Next, we validated the ODE model with experimental data, which was obtained from human primary pDCs activated with two different ligands [i.e., TLR7/8 ligand R848 and TLR9 ligand CpG-C], after which both the IFNα and TNFα production were monitored over time (26). The rationale for using freshly isolated pDCs over PBMCs boils down to the fact that PBMCs contain numerous cell types that will not produce IFN-I upon TLR7/8/9 activation, which could differ rather drastically in quantities from donor to donor. Their inhibitory role on the IFN-I system has yet to be fully characterized and could therefore introduce a significant amount of noise to our system, prompting us to use isolated pDCs instead. Accordingly, freshly isolated pDCs were activated with different concentrations of either R848 or CpG-C, over the course of 26 hours. The slight differences in concentrations used for R848 and CpG-C [i.e., 4 μg/mL R848 versus 5 μg/mL CpG-C] were based on differences in molecular weight and prior experience with the stimuli range [i.e., 100-fold] for proper pDC activation. R848 induced a very rapid and potent IFN-I production, which was dose independent (**Figure 2C**). However, the percentage of IFNα positive cells was highest for the lowest stimulus concentration [i.e., 0.4 μg/mL R848], but the difference was not significant, and was not reflected by the corresponding ELISA data, nor by the corresponding TNFα data (**Figure 2D**; **Supplementary Figure 2A**).

For CpG-C induced IFN-I signaling, a clear time-related, dose-dependent effect was observed, reflected by the highest stimulus concentrations initiating the fastest response (**Figure 2E**). Nevertheless, this reflected the lowest response of IFN-positive cells (**Figure 2E**). In contrast, lower concentrations resulted in slower responses, though characterized by the highest percentages of positive cells, and highest total secretion of IFNα (**Figure 2F**). On the contrary, regarding the TNFα production, we observed that the highest stimulus concentration initiated the highest response peaks, resulting in similar overall TNFα secretion (**Supplementary Figure 2B**).

To fit the model to the data, we first considered CpG-C stimulation at 0.5 and 5 μg/mL . Our goal was to find the most parsimonious change in parameters between the two dosages that is consistent with both the fraction of positive cells and secreted IFN α levels over time. Our results show that a change in two parameters – the threshold T=440 pg/mL and $k_{off}=0.2\;h^{-1}$ (for 0.5 μg/mL CpG-C) to $T=230\;pg/mL\;\text{and }k_{off}=0.33\;h^{-1}$ (for 5 μg/mL CpG-C) was sufficient to explain the data. All other parameters were unchanged between the two dosages and obtained as $k_{on}=0.4\;h^{-1}$ , the Hill coefficient H=4 $k_{f}=6600\;pg/mL/h$ and $d_{f}=0.04\;h^{-1}$ . Thus, a low threshold of activation of second responders in conjunction with faster turning off of IFN α positive cells with increasing stimulus dose leads to a faster kinetics of response but a lower peak fraction of second responders. Along the same trend, CpG-C stimulation at 50 μg/mL resulted in even a lower threshold of activation T=20 pg/mL and rapid turning off $k_{off}=2\;h^{-1}$ . Interestingly, data showed much lower levels of secreted IFN-I and required a reduction in the secretion rate to $k_{f}=2000\;pg/mL/h\;$ (from 6600 pg/mL/h for lower dosages) to capture the IFN α buildup. Finally, we fitted the model to data from R848 stimulation where we found the threshold for activation was similar to CpG-C stimulation at 50 μg/mL , but much faster kinetics of turning on $k_{on}=1.5\;h^{-1},\;$ and a tuning off rate $k_{off}=1\;h^{-1}$ that is faster than CpG-C stimulus at low dosages (0.5 and 5 μg/mL but slower as compared to 50 μg/mL CpG-C.

Accordingly, we conclude that increased CpG-C dosages decrease the threshold for second responder activation. In other words, a higher concentration of CpG-C can reach the activation threshold faster, as most likely the activation thresholds for the corresponding receptor [i.e., TLR9] remains the same across experimental conditions. Consequently, and verified by the modeling, increased CpG-C dosages result in the cells to turn off faster, explaining both the relatively low number of IFNα producing cells, and the relatively low level of total IFNα secreted. Interestingly, this difference in quantity was not observed for TNFα production, emphasizing that both cytokine systems are regulated differently. Together, these results show that TLR-7/8 and TLR9 induce remarkable different IFN-I response dynamics, with a clear dose-dependent effect upon TLR9 activation, which was not observed for TLR7/8 activation. The activation threshold for activating the second responders changed upon varying dosages targeting TLR9. Also, the rates of cells terminating IFN-I production changed, leading to fluctuations in second responder numbers throughout.

### 3.3 IFN-induced (de-)sensitization is noisy and subject to tight feedback regulation

Intrigued by the effects of varying TLR stimulation on IFN-I response dynamics, we set out to investigate the effects of IFN-priming on the population wide response dynamics, as both the crucial roles of paracrine signaling and the effects of IFN-induced (de-) sensitization have been described before (11, 14, 26, 36). Accordingly, a model-based analysis showed that prestimulation with a low IFNα dose hypersensitizes the IFN-I-pathway, whereas prestimulation with a high dose of IFNα leads to a dose-dependent desensitization (36). Similarly, we assessed the role of IFN-I priming on the percentages of IFNα-positive cells and the actual secretion of IFNα, reflected indirectly by mean fluorescence intensity (MFI) and directly by ELISA. To rule out additional extrinsic/intrinsic variation induced upon TLR stimulation, we chose to move forward with R848, as prior results indicated the lack of a dose-dependent effect, and the overall fast and potent activation of cells.

**Figure 3:**
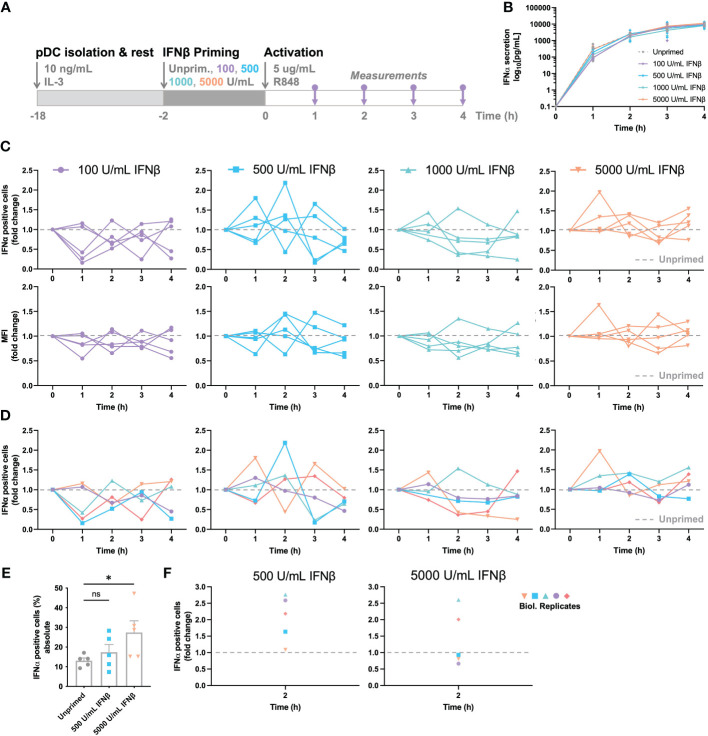
IFN-induced (de-)sensitization is noisy and subject to tight feedback regulation. **(A)** Schematic overview of experimental approach. pDCs were freshly isolated from buffy coats of healthy donors and rested overnight. Two hours prior to activation, pDCs were either primed with different concentration of IFNβ or left unprimed, in microwells containing 25.000 cells each. Cells were activated with 5 μg/mL R848. For the first 4 hours, for every hour the supernatant got collected for cytokine quantification and cells were fixed, permeabilized and stained for intracellular IFNα. **(B)** IFNα secretion over time. **(C)** IFNα response dynamics per different priming condition. Each connected line represents data from one donor. Data is depicted in fold change, based on the results from the unprimed conditions (dotted line). Both the percentages of IFNα positive cells (upper row), as the corresponding mean fluorescent intensities (MFIs; lower row) are depicted. **(D)** Data as in **(C)**, but color-coded by donor across the different priming conditions. **(E)** Absolute percentages of IFNα positive cells from one donor, divided over 5 biological replicates; mean ± SEM. **(F)** Data as in **(E)**, but depicted as fold change, based on randomized pairing with unprimed replicates. Student’s t-test *p< 0.05. ns, not significant.

In short, pDCs were isolated and rested overnight. Two hours prior to activation, cells were either left unprimed, or primed with different concentration of IFNβ, ranging from 100 to 5000 U/mL (**Figure 3A**). Thereafter, the cells were activated with R848 and monitored over the first four hours by intracellular IFNα staining and ELISA for cytokine quantification. Remarkably, IFN-priming does not significantly alter the total IFNα secretion over time, which is different from the sensitizing effects observed in a hepatocyte derived cellular carcinoma cell line upon priming with low dosages of IFN-I (**Figure 3B**) (36). A possible explanation for the difference with human primary pDCs could be that these cells rely on continuous self-priming by constitutive expression and signaling of IFN-Is, thereby overruling the additional priming effects induced in this experimental setup (37).

To unravel the effects of priming further, we next assessed the response dynamics per donor, thereby focusing on the IFNα positive cells and corresponding MFI of the IFNα positive events upon priming. To reveal the potential effects of (de-) sensitization, all primed conditions were compared to corresponding, donor-specific unprimed conditions, depicted by fold change. Accordingly, all values above 1.0 represent the effects of IFN-induced sensitization, whereas all values below 1.0 represent the effects of IFN-induced desensitization. Although the overall IFNα production was barely influenced by IFN-I priming, the response dynamics per donor provided some important insights into cellular decision-making and response dynamics. Remarkably, the two lowest priming concentrations of 100 and 500 U/mL IFNβ induced noisy dynamics, with conditions alternating massively between high (percentages over twice as high compared to the unprimed condition) and low responsiveness (percentages lower than half of the unprimed condition) over the course of the first four hours (**Figure 3C**). Similar phenomena were observed for the corresponding MFIs acquired by flow cytometry. This noisiness argues that cells are constantly probing their surroundings, thereby adjusting the population-wide response dynamics to avoid either too little or too high cytokine levels. Secondly, the two highest priming concentrations of 1000 and 5000 U/mL IFNβ induced seemingly less noisy dynamics, with less extreme differences in fold change values between hours, arguing that excessive levels of signaling molecules introduces less noise, perhaps by saturating all receptors and therefore inducing maximal effects, leaving less room for dynamic cellular decision-making. One hour priming showed rather similar fluctuations, though the higher priming concentrations seemed to induce more variation instead, indicating that, besides an important role for dosage, time is also an important factor in cellular decision-making (**Supplementary Figures 3A-D**).

Next, we aimed to correlate noisiness to individual donors by assessing the response dynamics for each donor, for the 4 different priming conditions. Interestingly, individual donors seemed to behave similarly across the 4 conditions, with some displaying high fluctuations over time, and other being less noisy (**Figure 3D**). To ensure that we are observing biological noise, rather than technological noise, we performed the same procedure on 5 biological replicates. In other words, for testing the biological noise, the same experimental conditions were performed in parallel 5 times, using freshly isolated pDCs from the same donor. For testing the technical noise, one experimental condition was handled, stained, and measured 5 times. The technological noise, as well as the unprimed conditions of the biological noise test appeared to give a relatively similar data spread (SD of 2.624 and 3.013 respectively; **Figure 3E**). In contrast, the primed conditions, although from the same donor, gave rather large fluctuating percentages of IFNα-positive cells (SD of 8.737 and 13.257 for 500 and 5000 U/mL IFNβ respectively). Upon transformation to fold change values, based on random matching of unprimed and primed replicates, the data within a donor is as noisy as between donors (**Figure 3F**). Therefore, we conclude that the IFN-I system is inherently noisy, arguing important roles for both positive and negative feedback loops.

Altogether, we conclude that IFN-induced (de-)sensitization is noisy and subject to tight feedback regulation, without having an overall quantitative effect on total IFN-I production in human primary pDCs. However, over the course of the IFN-I production, especially at low priming dosages, cells seem to fluctuate, thereby tune their production throughout. Of note, the fraction of second responders seems to fluctuate both within and across donors, suggesting that these two different cell fates rely on different underlying regulatory mechanisms.

### 3.4 Blocking TNFα signaling increases IFNα production

After having characterized the basics on IFN-I and TNFα response dynamics in human primary immune immune cells, we aimed to move forward to studying their crosstalk. Especially in the field of autoimmunity, understanding this crosstalk is of high relevance, as multiple treatments are relying on inhibiting one of the two systems specifically (e.g., Adalimumab or Upadacitinib for blocking either TNFα or primarily IFN-I signaling, respectively). Nevertheless, a clear understaning on the consequence of inhibiting only one system on the untargeted cytokine system remains limited. Moreover, previous studies have mainly explored these concepts in bulk cultures, averaging out potential heterogeneous cellular behaviors (30, 31). Besides a fundamental conceptualization, additional insights can also be considered clinically relevant. For example, to treat RA, treatment strategies often rely on blocking TNFα or IL-6, while it is widely appreciated that the IFN-I system is often aberrant too (3). Whether this is a result of the conventional treatments, or whether this is an intrinsic disease characteristic remains to be further elucidated.

**Figure 4:**
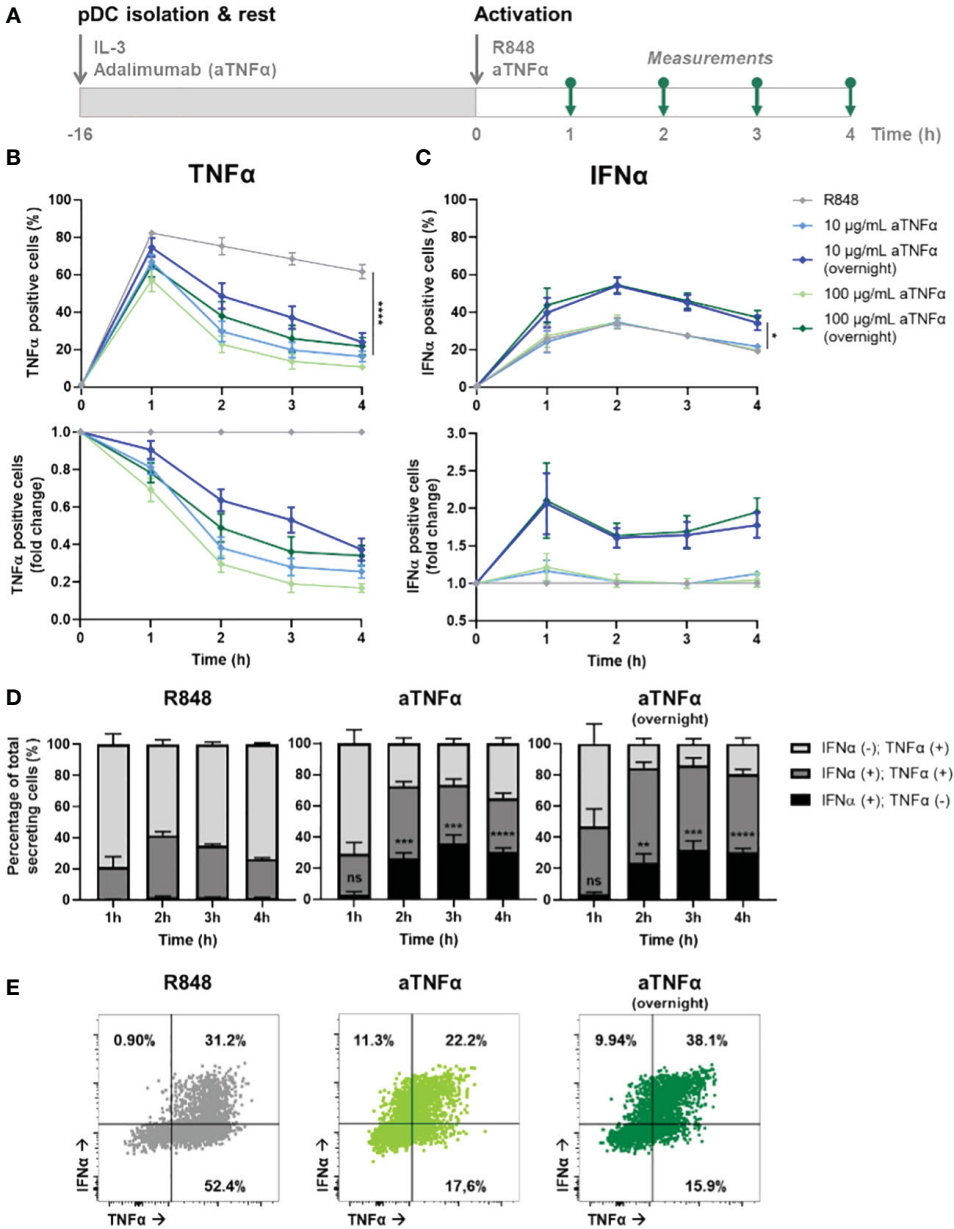
Dynamic TNFα and IFNα crosstalk and co-expression upon TNFα blocking. **(A)** Schematic overview of experimental approach. pDCs were freshly isolated from buffy coats of healthy donors and rested overnight, and either pre-incubated overnight with anti-TNFα (aTNFα) or left untreated in microwells containing 25.000 cells each. Next, cells were activated with 5 μg/mL R848. For the first 4 hours, for every hour cells were fixed, permeabilized and stained for intracellular TNFα and IFNα. **(B)** TNFα response dynamics, depicted by absolute percentages of TNFα positive cells and fold change over untreated conditions. **(C)** IFNα response dynamics, depicted by absolute percentages of IFNα positive cells and fold change over untreated conditions. **(D)** Bar graphs depicting the co-expression of IFNα and TNFα, based on single and double positivity, for the different experimental conditions. **(E)** Corresponding dot plots for t = 2h, as in **(D)**. n = 5; Mean ± SEM; Mixed-effects analysis followed by a Bonferroni’s multiple comparisons test *p< 0.05, **p< 0.01, ***p< 0.001, ****p< 0.0001. ns, not significant.

Although studying the fundamentals of cellular-decision making during IFN-I and TNFα signaling in patients could enhance the translatability of the results, a challenge in interpretation of the results might lie in the possibility that a difference in medication history affects the inherent secretory behaviors of their immune cells. Instead, by characterizing the effects of blocking one immune signaling system in cells from healthy donors, this noise-introducing factor will be excluded. Following this reasoning, we isolated primary pDCs from healthy donors, incubated the cells with or without anti-TNFα (aTNFα, adalimumab) overnight (pre-incubation), and activated the cell with R848 the following day (**Figure 4A**). Besides the pre-incubation with aTNFα, additional experimental conditions contained pDCs that were treated with aTNFα and activated with R848 simultaneously, referred to as co-incubation.

The results show a dose-dependent effect of aTNFα on inhibiting TNFα production, quantified by the percentage positive cells (**Figure 4B**). Besides, also the amount of cytokine production per cell was inhibited, reflected by the corresponding mean fluorescent intensities (MFI; **Supplementary Figure 4A**). Remarkably, pre-incubated cells show a less decrease in percentage of positive cells and corresponding MFI values, compared to the untreated cells. This implies that the cells can become desensitized to the treatment, meaning that the pre-incubation of aTNFα can lead to diminished functional effects. In contrast, the effects of treatment regarding the IFN-I production are only observed upon pre-incubation, leading to up to twice as high number of IFNα positive cells, while co-incubation does not cause a significant difference (**Figure 4C**). The corresponding MFI values did not change for either of the conditions, meaning that the effects of aTNFα only influence the number of cells becoming producers, rather than the actual cytokine production per cell (**Supplementary Figure 4B**).

Next, we wondered how the treatment would affect co-expression of TNFα and IFNα in individual cells. Upon R848 activation, roughly all cells that become IFNα positive are also TNFα positive (**Figure 4D**). Therefore, it is likely to assume that TNFα is produced first, and from this pool of cells also the IFN-I producers arise. This is in agreement with studies proving a pool of constitutively transcribed TNF mRNA being present in macrophages, thereby explaining the ability of a cell to start producing TNFα faster than IFN-I (38). Upon treatment with aTNFα, either pre- or co-incubation, events that are only positive for IFNα appear, but only after two hours (**Figure 4E**; **Supplementary Figure 5**). This would still allow the IFNα positive events to arise from TNFα producing cells, however, the TNFα secretion gets diminished upon blocking the autocrine/paracrine feedback loops, leaving the production of IFNα unaffected.

Altogether, we provided additional proof for an IFN–TNFα crosstalk, which is affected by inhibiting TNFα, resulting in additional IFN-I production. Therefore, we hypothesize an inhibitory effect of TNFα on IFN-I production, which is lifted upon blocking TNFα signaling. Additionally, TNFα secretion enhances additional TNFα production, likely both in an autocrine and paracrine fashion, which holds also true for the IFN-I system.

### 3.5 Blocking IFNα signaling decreases TNFα production

After having validated the presence of a potent IFN–TNFα crosstalk by blocking TNFα signaling, we wondered whether we could observe similar effect upon blocking primarily IFN-Is instead. Similar to the clinical relevance of blocking TNFα to treat RA, JAK inhibitors (JAKi) are increasingly getting prescribed, as multiple studies report their promising effects in treating RA (39). Of note, JAKi Upadacitinib is a selective JAK1 inhibitor, which is not specific to the IFNAR receptor. Instead JAK1 is part of numerous type I and II cytokine receptors, therefore affecting numerous cytokines (e.g., IL-2, IL-6, IL-10, IFN-gamma, etc.). However, in our experimental settings using pDCs, which dedicate over 50% of their transcriptome to IFN-I production upon activation, we assume that IFN-Is are the main target (40).

**Figure 5:**
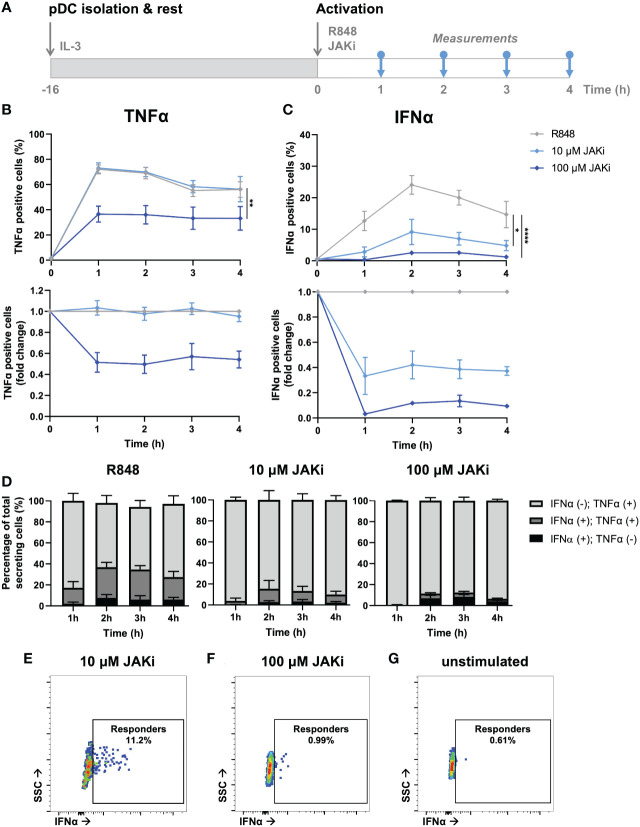
Dynamic TNFα and IFNα crosstalk and co-expression upon IFN-I blocking. **(A)** Schematic overview of experimental approach. pDCs were freshly isolated from buffy coats of healthy donors and rested overnight. Next, cells were activated with 5 μg/mL R848 and incubated with 10 or 100 μM JAKi. For the first 4 hours, for every hour cells were fixed, permeabilized and stained for intracellular TNFα and IFNα. **(B)** TNFα response dynamics, depicted by absolute percentages of TNFα positive cells and fold change over untreated conditions. **(C)** IFNα response dynamics, depicted by absolute percentages of IFNα positive cells and fold change over untreated conditions. **(D)** Bar graphs depicting the co-expression of IFNα and TNFα, based on single and double positivity, for the different experimental conditions. **(E)** Corresponding dot plot of biological replicate of cells activated with R848 for 2h, treated with 10 uM JAKi. **(F)** Corresponding dot plot of biological replicate as in **(E)**, treated with 100 uM JAKi. **(G)** Corresponding dot plot of biological replicate as in **(E)**, however unstimulated and untreated. n = 5; Mean ± SEM; Mixed-effects analysis followed by a Bonferroni’s multiple comparisons test *p< 0.05, **p< 0.01, ****p< 0.0001.

From a mechanistic point of view, we hypothesized that upon incubation with JAKi (Upadacitinib), IFN-I production could only be initiated in first responders, as by blocking JAK1 (downstream of the IFNAR) the effects of paracrine signaling gets blocked (41). To study this, we isolated, rested overnight, and activated pDCs with varying JAKi concentrations (**Figure 5A**). Upon incubation with 10 μM JAKi, the TNFα secretion dynamics did not change (**Figure 5B**). In contrast, the production of IFNα was largely inhibited, though not completely to the percentages which we considered similar to the fraction of first responders (~10% versus 1-3% first responders) (**Figure 5C**). Therefore, we concluded that at this concentration not all IFNAR signaling was successfully blocked. By increasing the concentration 10-fold (100 μM), we hypothesized to successfully saturate, and thereby block all IFNAR signaling. Indeed, the percentages of IFNα producing cells was diminished to similar levels as the fraction of first responders usually present (1-3%). Interestingly, it also drastically influenced the secretion of TNFα, arguing that, during physiological conditions, part of the TNFα is dependent on paracrine-induced IFN-I signaling. Similar to the results obtained upon aTNFα treatment, the MFI values obtained upon JAKi treatments displayed similar trends compared to the percentages of cytokine secreting cells (**Supplementary Figures 6A, B**).

Similar to the co-expression analysis performed upon TNFα blocking, we performed a similar analysis on the data obtained upon IFN-I blocking. In contrast to the effects of TNFα blocking on co-expression, IFNα positive cells treated with JAKi were most of the time also positive for TNFα, especially upon treatment with 10 μM (**Figures 5D, E**; **Supplementary Figure 7**). As the percentages of positive cells upon treatment with 100 μM JAKi were very small, it is statistically less reliable to draw conclusions from the co-expression of TNFα and IFNα under these experimental conditions, although the trend appeared that the numbers for IFNα(+);TNFα(+) and IFNα(+);TNFα(-) are about equal. This would indicate that, in this particular situation, the IFN-I production is independent from TNFα production.

Finally, we checked whether the rather small percentages of IFNα positive cells upon treatment with 100 μM JAKi could be considered genuine first responders, instead of the positive events simply reflecting fluorescent or biological noise. Interestingly, the events that were considered IFNα positive could clearly get distinguished from the fluorescent and biological noise, as compared to the unstimulated control, but their MFI was noticeably lower than those obtained in the 10 μM condition (**Figure 5F, G**). This strengthens the argument that these events represent only first responders, as also these cells need IFNAR signaling (via autocrine signaling) to enhance their own IFNα production. Likewise, cells treated with 10 μM JAKi are still able to enhance their own IFNα secretion upon autocrine signaling, and to initiate IFNα secretion in a small fraction of surrounding cells, upon paracrine signaling.

In short, blocking IFNAR signaling only influenced TNFα response dynamics at saturating dosages, resulting in lower TNFα production. As it did not completely halt TNFα production, we conclude that the IFN-I system only partly affects the TNFα system, possibly by only enhancing secondary TNFα production overtime. In other words, certain levels of TNFα can be produced independent on IFN-I signaling, whereas increasing levels of TNFα producing are dependent on IFN-I signaling.

### 3.6 Aberrant TNFα and IFN-I response dynamics in SLE patients

SLE is one of many autoimmune diseases associated with aberrant IFN-I regulation (3, 42). As SLE is characterized by a prominent expression of IFN-stimulated genes in 50%-75% of adult patients, this disease was of highest interest to assess whether the pathology involves a dysregulated IFNα–TNFα crosstalk (43). What exactly induces the increased IFN-I production in the majority of SLE patients remains mysterious. However, numerous possible inducers and regulators of the excessive IFN-I production in pDCs have been hypothesized (44).

**Figure 6:**
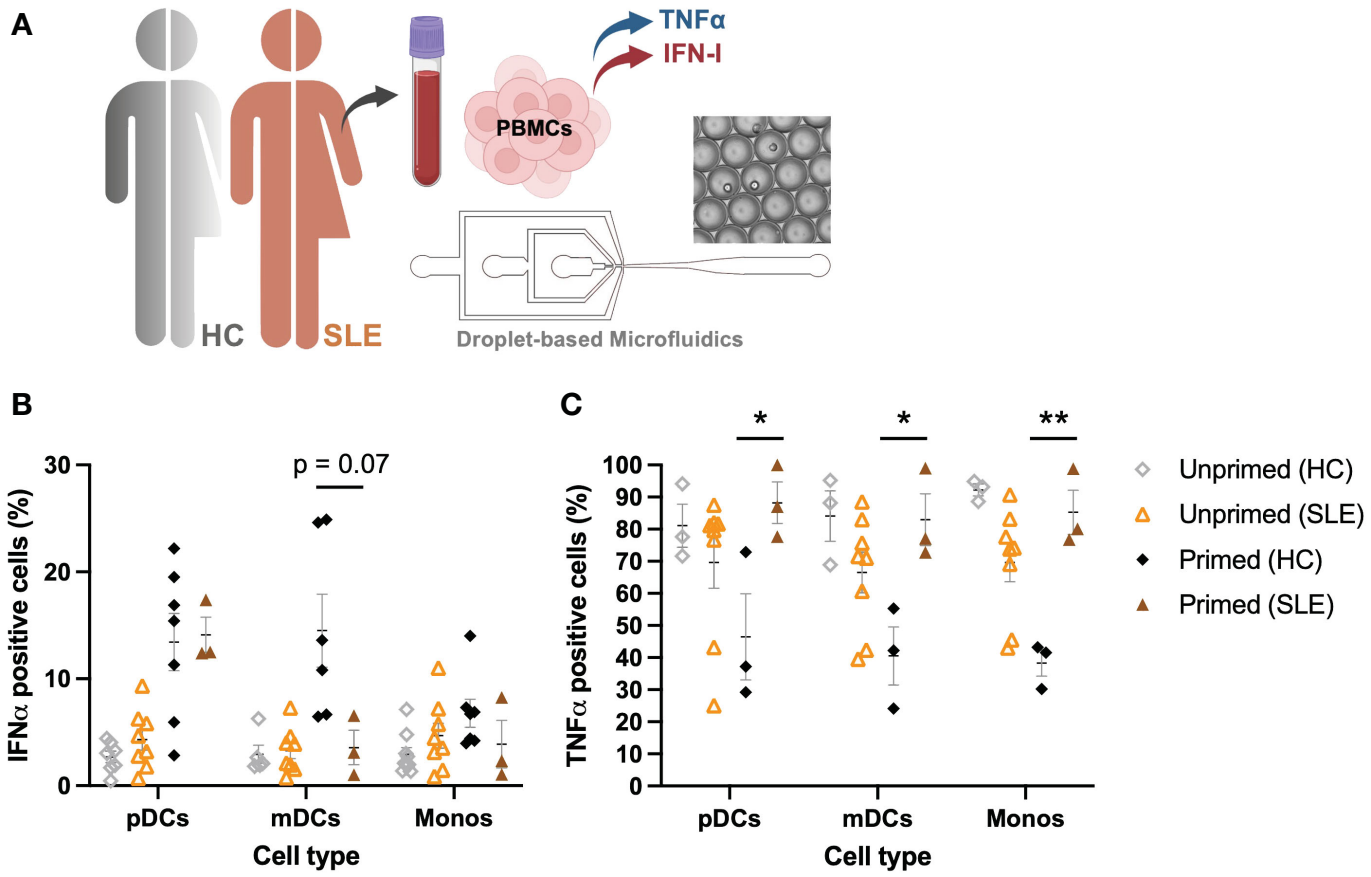
Aberrant IFNα–TNFα crosstalk in SLE patients. **(A)** Schematic overview of experimental approach. PBMCs from SLE patients (n=8) were isolated and encapsulated in droplets like described earlier. **(B)** Percentages of IFNα positive cells, unprimed compared to primed (500 U/mL IFNβ, 2h), upon single-cell activation with 50 μg/mL R848. Data of HCs as in **Figure 1D**. **(C)** Percentages of TNFα positive cells, unprimed compared to primed (500 U/mL IFNβ, 2h), upon single-cell activation with 50 μg/mL R848. Data of HCs as in **Figure 1E**. Mean ± SEM; Student’s t-test * p< 0.05, **p< 0.01.

For this study, 8 SLE patients were included in the cohort, of which PBMCs were isolated from whole blood (**Figure 6A**). Using the droplet-based microfluidics platform, PBMCs obtained from patients were encapsulated in droplets to assess their intrinsic secretory behaviors upon single-cell activation, as described before. Interestingly, pDCs obtained from SLE patients showed similar secretory behaviors, with a rather small fraction of first responders (mean 4.32 +/- 2.79 SD, compared to mean 2.41 +/- 1.30 SD in HC; p = 0.15, unpaired t test) and an increased compared to unprimed, but again similar fractions of second responders between SLE and HCs upon priming (mean 14.1 +/- 2.86 SD, compared to mean 13.44 +/- 7.10 SD in HC; p = 0.88, unpaired t test) (**Figure 6B**). In contrast, both mDCs and monocytes displayed no significant effects of priming, meaning both primed and unprimed conditioned gave similar numbers of IFNα producing cells.

Whereas PBMCs from HCs were all capable of producing TNFα in droplets upon single-cell activation, PBMCs obtained from SLE patients showed decreased percentages of TNFα positive cells (**Figure 6C**). In stark contrast to what was observed in HCs (where priming led to a decrease in TNFα positive cells), priming cells obtained from SLE patients significantly increased the number of TNFα positive cells. In short, mainly the TNFα system seems to be dysregulated in these patients, whereas the IFN-I system seems to behave considerably similar to the system in HCs, of note, under physiological conditions. However, besides the increased IFN-I signatures that are often associated with SLE, serum TNFα levels are often elevated too, showing a positive correlation between serum TNFα and IFNα (45). This is in contrast with the physiological conditions in which the two different cytokine types inhibit each other (30). Therefore, understanding the interplay between these two major cytokine groups in pathological conditions is key in further unraveling the pathogenesis of SLE and other autoimmune diseases affected by aberrant cytokine signaling.

In conclusion, the increased IFN-I signature observed in 50%-75% of SLE patients is more likely induced by an increased trigger of IFN-I production, rather than aberrant cellular-decision making upon IFN-I signaling. Therefore, the current hypothesis that pDCs are largely held responsible for the ongoing IFN-I production in SLE could still hold true, but perhaps only because they are triggered to greater extend, rather than being hypersensitive. Interestingly, a recent study on cutaneous lupus points away from pDCs being the major IFN-I producers (46).

## 4 Discussion

Cellular decision-making exhibits different levels of heterogeneity originating from variations in genome architecture (47, 48) in concert with regulatory signaling events (49, 50), through intrinsic noise in stochastic processes and extrinsic differences between cells (51–53). In other words, single-cell gene expression is inherently variable, but how this variability is controlled on the population level remains largely elusive. To decipher this, we used droplet-based microfluidics and mathematical models to explain single-cell secretory behaviors for both IFN-I and TNFα systems. We addressed their potential crosstalk by blocking either one of the two signaling systems, which revealed that the two systems are intertwined. Regarding the current approaches for treating autoimmune diseases, which mainly target either one of the systems, it would be of great importance to further unravel the crosstalk between the two pro-inflammatory cytokines to both personalize treatments and predict the effectiveness of the treatments. Mathematical modeling could be a useful tool to achieve that, as once the models are validated, one is able to intervene (mimicking treatment) and assess the outcome (predicting the clinical outcome).

Whereas the concept of first, second, and non-responders is still relatively new, our findings provide additional proof of the phenomenon holding true for IFN-I-secreting human primary immune cells, which goes beyond the already well-established proof in pDCs. These findings further emphasize that the phenomenon occurs across different cell types, indicating that it can be intrinsic to the IFN-I system. Of note, while IFNβ priming induced increased IFN-I producing cells upon single-cell activation, the roles of other cytokines, especially those induced upon IFNAR signaling, in dictating IFN-I response dynamics still need to be explored. Another important question which remains unanswered is, not only how, but also when these fates [i.e., first, second, and nonresponders] are assigned to the immune cells, as this opens new treatment approaches. For example, if these fates are assigned already in the bone marrow, while originating from the hematopoietic progenitor cells, one can think of treatments targeting the hematopoietic progenitor cells to reassign cell fate. In contrast, if the fates. are assigned at a later phase in immune cell development, for example, once in the periphery, one can think of a systemic treatment approach to coordinate immune cell fates.

From a more fundamental point of view, it is interesting to speculate on why the two cytokine systems are regulated as differently as observed in this study. As the harmful effects of excessive levels of IFN-Is are well appreciated, it seems logical that this system must be more carefully regulated, which could explain the clear distinction between responders and non-responders, with the additional layer of first and second responders. Perhaps excessive levels of TNFα are less harmful, and therefore tolerate a less complicated regulation, making it less prone to mistakes. In other words, pro-inflammatory signaling systems and their internal control of magnitude are dictated by a tradeoff between susceptibility to infection and to immunopathology, which might be different for the two systems (5). To further explore the differences, TNFα priming (in both HCs and SLE), instead of IFN-I priming can provide additional insights into the effects of excessive levels of TNFα and its effects in health and disease.

Not only are the two cytokine systems regulated differently, but even within the IFN-I system we observed different responses. In particular, the rather fixed fraction of first responders, and the fluctuating fraction of second responders argue that these two cell fates are regulated differently. We currently hypothesize that the fraction of first responders is predetermined, dictated by rare, transiently heritable cell variability, as observed as a mode for cancer drug resistance and genetic programs associated with IFN-I signaling (54, 55). In contrast, we currently hypothesize that the fraction of second responders is stochastically regulated. Accordingly, stochastic control allows for cellular heterogeneity that has benefits over hard-wired deterministic cell fates, both at the level of initiation and outcome response (56). Especially considering the wide variety of pathogens, the immune system must encounter, a fully deterministic strategy is far from ideal, as many different types of immune cells are involved, and the combinations of inputs are unpredictable. However, having a reliable first burst of IFN-Is produced by the first responders is crucial to dictate population-wide response dynamics, and could therefore rely on deterministic principles (14).

Over millions of years, immune systems have evolved by balancing out the tradeoffs posed by infection and immunopathology (5, 57). However, as these tradeoffs are not necessarily symmetrical, theoretically, natural selection favors strong defenses, with overshooting (producing excessive levels of proinflammatory cytokines) at risk. Autoimmune diseases are the perfect example of such catastrophic overshooting, during which the immune system gets activated without the need to fight a potential threat, or more generally, perpetuating inflammation in a pathogenic, way as observed in autoimmunity triggered by cancer (3, 8, 58–60). Accordingly, in SLE patients, the IFN-I system seems to be dysregulated, characterized by elevated levels of systemic IFN-I signaling (reviewed in (44). However, our results indicate that the fundamentals of single-cellular decision-making leading to IFN-I production in pDCs are not necessarily different from pDCs isolated from healthy donors, giving similar percentages of IFN-I producing cells. This contrasts with earlier studies describing that pDCs are functionally impaired in SLE and cutaneous lupus (46, 61). Of note, these findings were obtained in bulk-activated pDCs, which allows for cellular communication that could lead to either an overall decrease in IFN-I production, or different secretion dynamics that are not captured timely. Droplet-based microfluidics overcomes both, while paracrine signaling is no longer physically possible, and secretion dynamics are captured over longer periods of time while secreted cytokines accumulate in the droplets. In contrast to the pDCs, in our hands, a smaller fraction of mDCs and monocytes produced IFN-I upon paracrine signaling and TLR stimulation, compared to cells obtained from healthy donors. Future studies should further validate these findings, while our patient cohort was rather small.

Overall, SLE is a very heterogeneous disease (44). Generally, the importance of IFN-I in the pathogenesis of SLE has been accepted based on the observed IFN-I gene signatures in the majority of patients, as well as success of IFN-blocking therapy in phase III clinical trials (62). However, the source of IFN-Is are still under debate. Endogenous nucleic acids and their interactions with autoantibodies have been proposed to be the main stimulator of pDCs (63–66). Accordingly, pDCs were naturally assumed to be the main source of IFN-Is in SLE, while recent literature brings uncertainty to this assumption. Unsorted PBMCs obtained from SLE patients have been shown to produce lower levels of IFN-I *in vitro*, while in contrast other studies reported enhanced IFN-I production by pDCs obtained from SLE patients (67, 68). These contradicting results can be explained by (often minor) differences in methodologies and experimental approaches. Especially experiments performed in bulk allow for variation, while heterogeneous subsets can drive population outcomes. Using droplet-based microfluidics, (immune-) cells can be activated in a highly controlled manner, eliminating the influence of external, often uncharacterized effects. Regarding our results obtained in pDCs, we hypothesize that the pathophysiology of SLE, and perhaps other autoimmune diseases affected by aberrant IFN-I signaling (e.g., RA), lies not in the dysfunctional inherent abilities of pDCs responding to cues and triggers. Instead, a variety of triggers unrelated to fighting pathogens have been associated with the excessive production of IFN-Is in SLE patients (44). Reducing these triggers might be a better approach to correct for the aberrant IFN-I response dynamics, instead of targeting IFN-Is, or pDCs directly. Finally, as indicated by our results, one should realize that targeting a certain cytokine system (e.g., IFN-I signaling) is likely also affecting other cytokine systems (e.g., TNF signaling), perhaps with unintended consequences. Droplet-based microfluidics, and other elegant engineering approaches, in combination with modeling, allows for further characterization of such interactions.

## Data availability statement

The raw data supporting the conclusions of this article will be made available by the authors, without undue reservation.

## Ethics statement

All patients provided informed written consent approved by the local institutional medical ethics review boards prior to inclusion in this study (NL47151.041.13). The studies were conducted in accordance with the local legislation and institutional requirements. The participants provided their written informed consent to participate in this study.

## Author contributions

LE: Conceptualization, Formal analysis, Investigation, Methodology, Writing – original draft, Writing – review & editing. EC: Conceptualization, Formal analysis, Investigation, Writing – review & editing. CM: Investigation, Writing – review & editing. AP: Conceptualization, Writing – review & editing. TR: Conceptualization, Writing – review & editing. JB: Conceptualization, Resources, Writing – review & editing. AS: Conceptualization, Formal analysis, Investigation, Methodology, Writing – original draft, Writing – review & editing. JT: Conceptualization, Funding acquisition, Supervision, Writing – review & editing.
