# Supplementary material for: Unraveling IFN-I response dynamics and TNF crosstalk in the pathophysiology of systemic lupus erythematosus
Source: Front Immunol. 2024 Mar 26;15:1322814. doi: 10.3389/fimmu.2024.1322814 (PMC11002168; doi:10.3389/fimmu.2024.1322814)
Supplement: Supplementary file 1 [file DataSheet_1.docx]

Supplementary Material

**
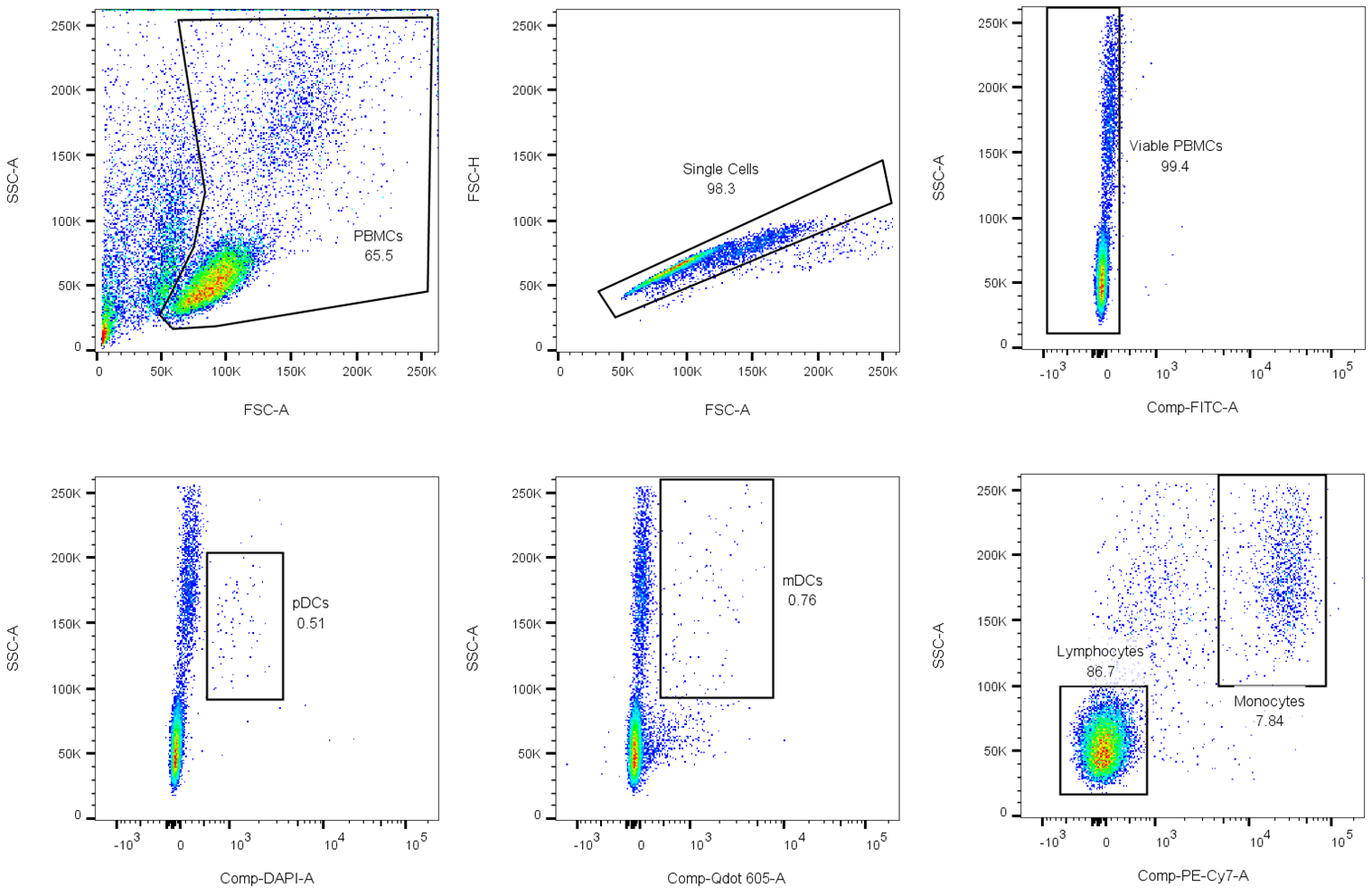
**

**Supplementary Figure 1: PBMC gating strategy.** Total PBMCs were gated based on morphology (using FSC-A and SSC-A readouts), after which the singlets were defined using FSC-H. PBMCs stained by Zombie Green (viability dye) were excluded from the analysis by defining the viable PBMCs for all negative events. pDCs were gated according to the expression of CD123–BV421 (DAPI detector), mDCs according to the expression of CD1c–BV605 (Qdot 605 detector), and monocytes according to the expression of CD14–PE-Cy7. All subset gates were defined according to both marker expression and SSC-A as a guideline for cell size.

**
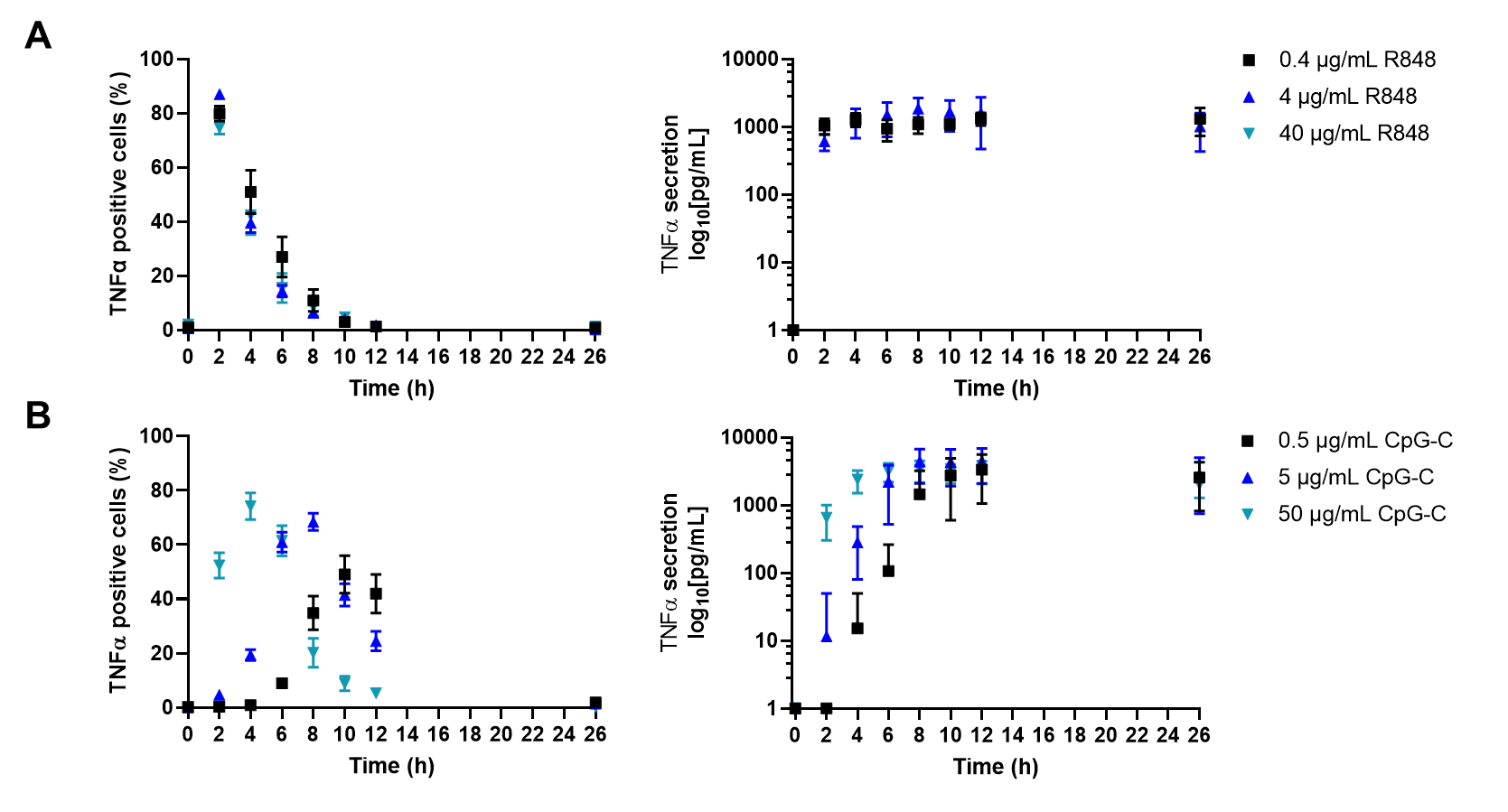
**

**Supplementary Figure 2: TNFα response dynamics upon varying TLR stimuli. (A)** TNFα response dynamics, represented as percentage of positive cells (left panel), and corresponding TNFα secretion dynamics (right panel) upon various concentrations of R848; mean ± SEM; n = 4-14 and 5-7, for left and right panel, respectively. **(B)** TNFα response dynamics, represented as percentage of positive cells (left panel), and corresponding TNFα secretion dynamics (right panel) upon various concentrations of CpG-C; mean ± SEM; n = 5-14, for both panels.

**
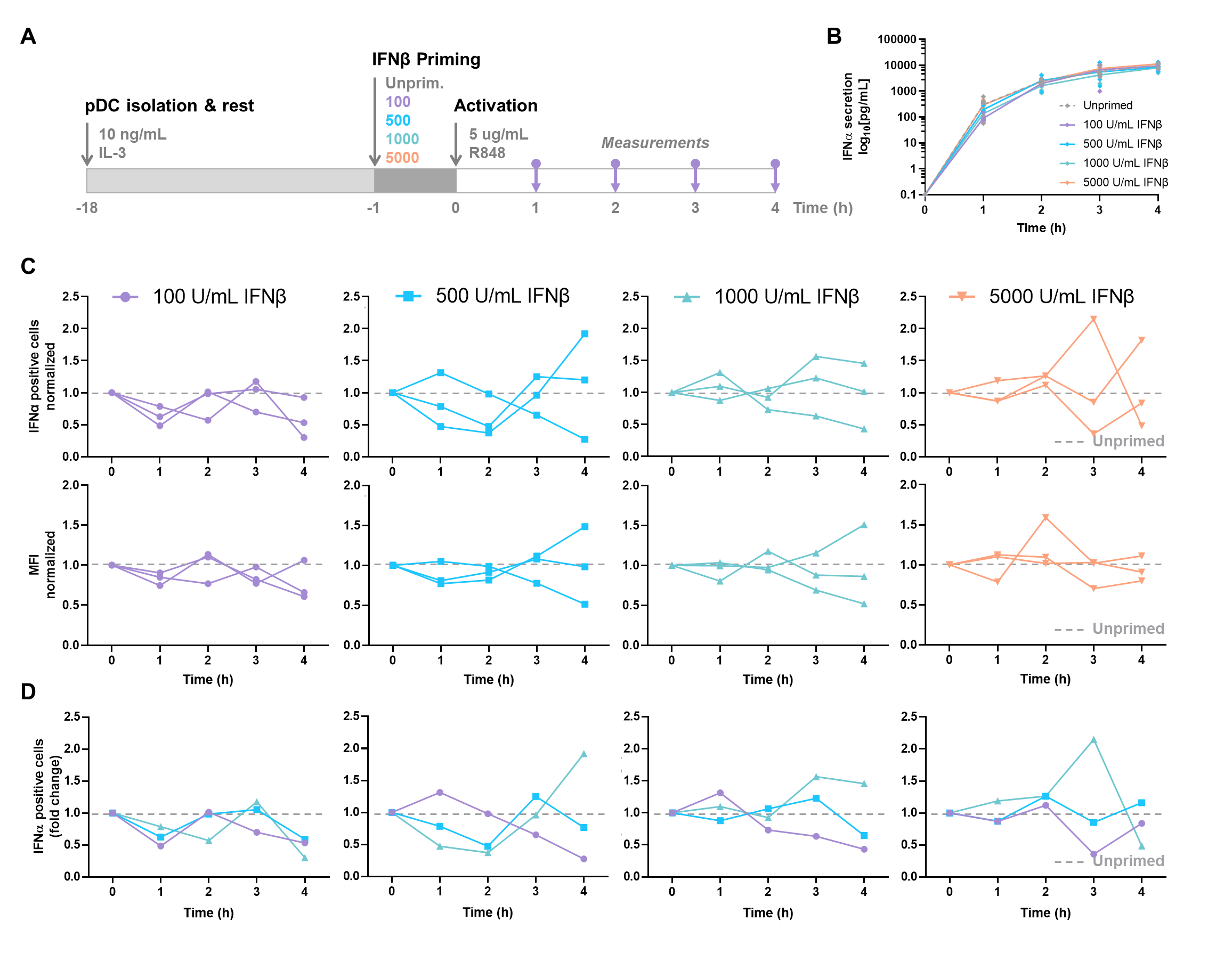
**

**Supplementary Figure 3: IFN-induced (de-)sensitization after 1h IFNβ priming. (A)** Schematic overview of experimental approach. pDCs were freshly isolated from buffy coats of healthy donors and rested overnight. One hours prior to activation, pDCs were either primed with different concentration of IFNβ or left unprimed, in microwells containing 25.000 cells each. Cells were activated with 5 μg/mL R848. For the first 4 hours, for every hour the supernatant got collected for cytokine quantification and cells were fixed, permeabilized and stained for intracellular IFNα. **(B)** IFNα secretion over time. **(C)** IFNα response dynamics per different priming condition. Each connected line represents data from one donor. Data is depicted in fold change, based on the results from the unprimed conditions (dotted line). Both the percentages of IFNα positive cells (upper row), as the corresponding mean fluorescent intensities (MFIs; lower row) are depicted. **(D)** Data as in **(C)**, but color-coded by donor across the different priming conditions.

**
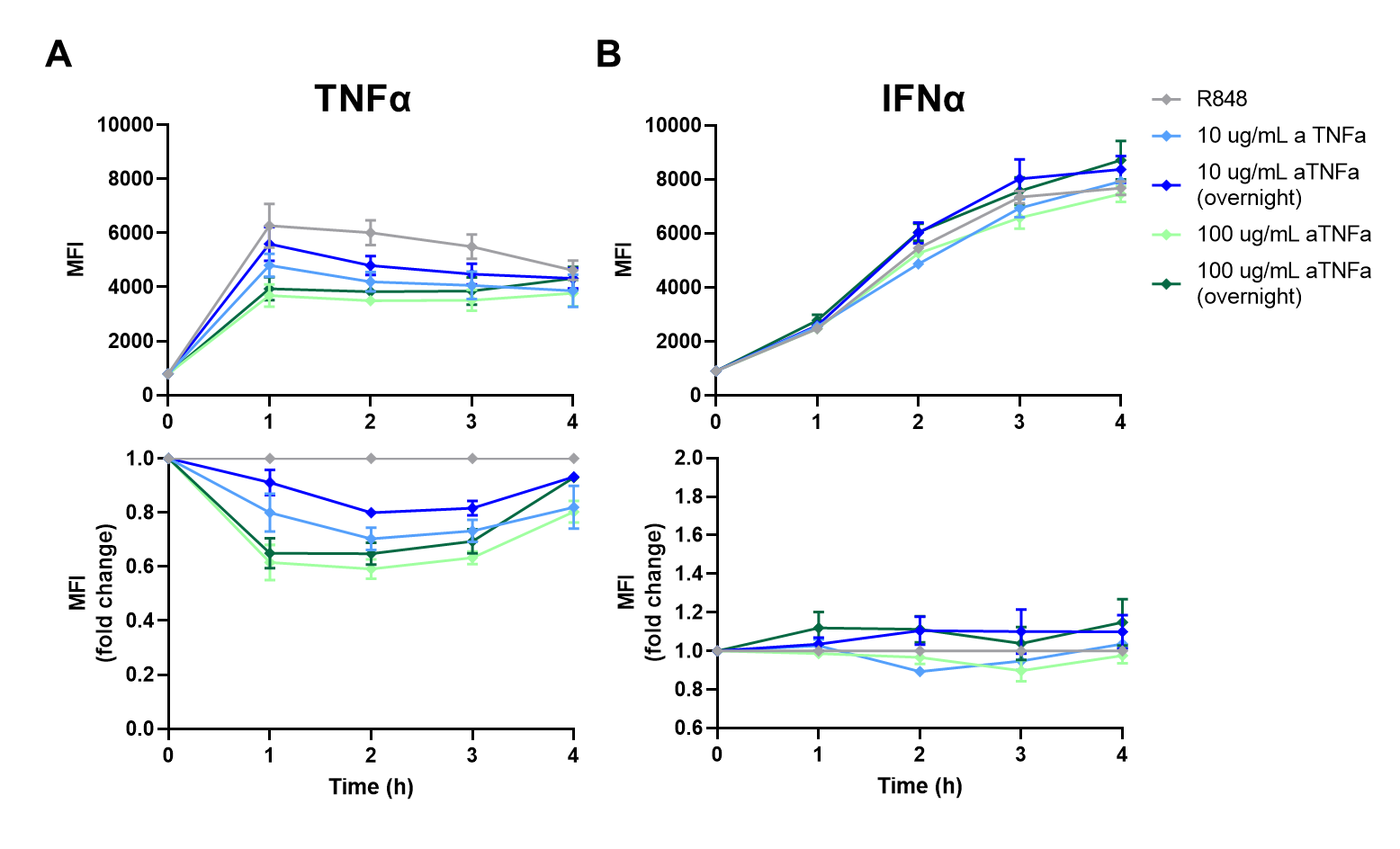
**

**Supplementary Figure 4: MFI values upon anti-TNFα (aTNFα) treatment.** pDCs were freshly isolated from buffy coats of healthy donors and rested overnight, and either incubated overnight with anti-TNFα (aTNFα) or left untreated in microwells containing 25.000 cells each. Next, cells were activated with 5 μg/mL R848. For the first 4 hours, for every hour cells were fixed, permeabilized and stained for intracellular TNFα and IFNα. **(A)** MFI values of TNFα production, depicted by absolute number (upper panel) and fold change (lower panel). **(B)** MFI values of IFNα production, depicted by absolute number (upper panel) and fold change (lower panel).


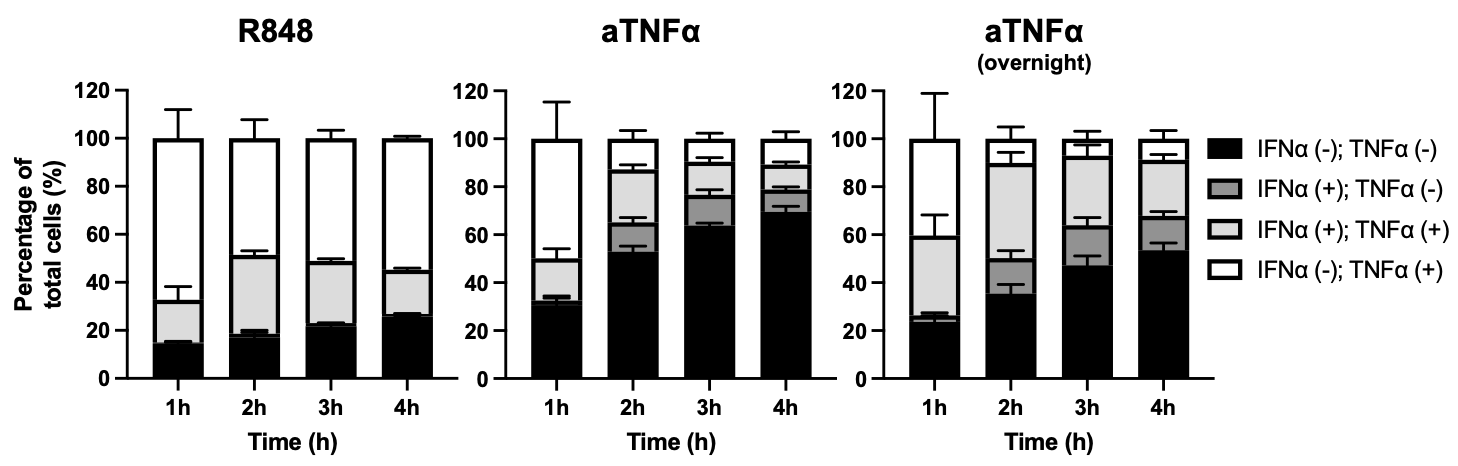


**Supplementary Figure 5: Dynamic TNFα and IFNα crosstalk and co-expression upon TNFα blocking.** Bar graphs depicting the co-expression of IFNα and TNFα, as in Figure 4D, now including double negative populations. Depicted are the mean ± SD.

**
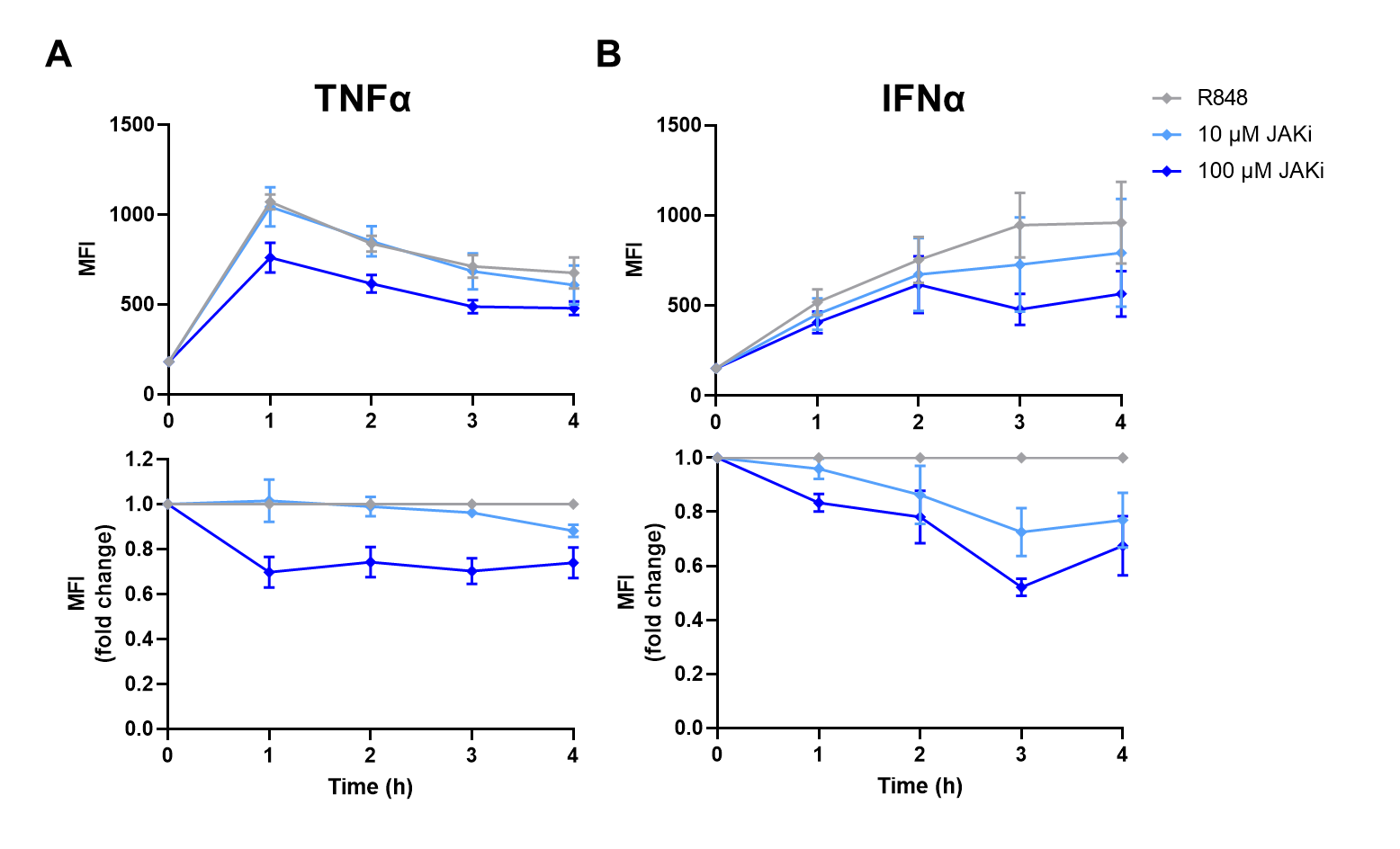
**

**Supplementary Figure 6: MFI values upon JAKi treatment.** pDCs were freshly isolated from buffy coats of healthy donors and rested overnight in microwells containing 25.000 cells each. Next, cells were activated with 5 μg/mL R848 and treated with JAKi. For the first 4 hours, for every hour cells were fixed, permeabilized and stained for intracellular TNFα and IFNα. **(A)** MFI values of TNFα production, depicted by absolute number (upper panel) and fold change (lower panel). **(B)** MFI values of IFNα production, depicted by absolute number (upper panel) and fold change (lower panel).


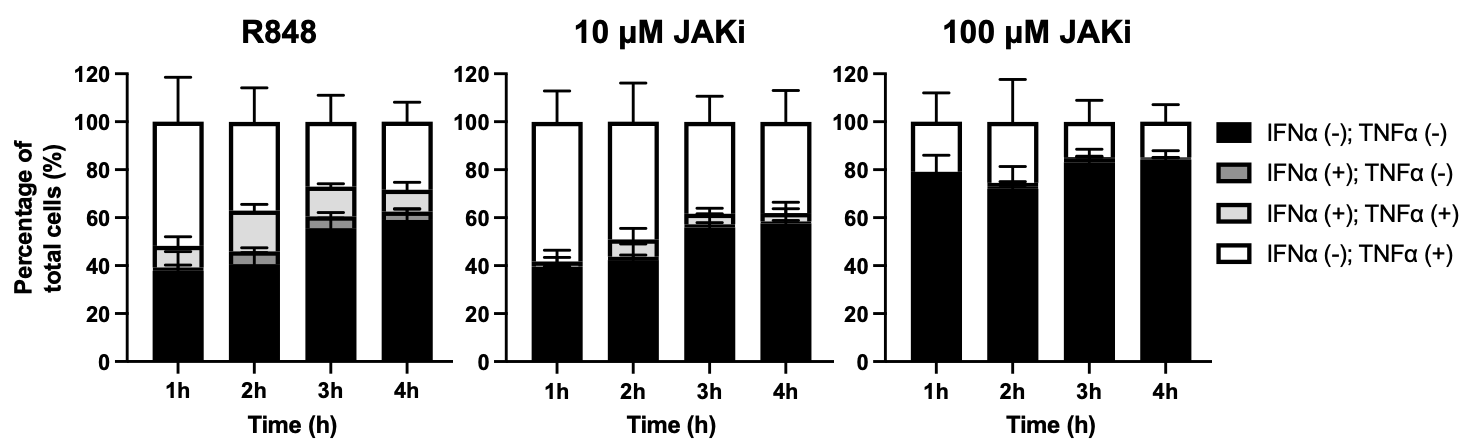


**Supplementary Figure 7: Dynamic TNFα and IFNα crosstalk and co-expression upon IFN-I blocking.** Bar graphs depicting the co-expression of IFNα and TNFα, as in Figure 5D, now including double negative populations. Depicted are the mean ± SD.
